# Marine N-3 Polyunsaturated Fatty Acids Are Inversely Associated with Risk of Type 2 Diabetes in Asians: A Systematic Review and Meta-Analysis

**DOI:** 10.1371/journal.pone.0044525

**Published:** 2012-09-11

**Authors:** Ju-Sheng Zheng, Tao Huang, Jing Yang, Yuan-Qing Fu, Duo Li

**Affiliations:** 1 Department of Food Science and Nutrition, Zhejiang University, Hangzhou, China; 2 APCNS Centre of Nutrition and Food Safety, Hangzhou, China; Postgraduate Medical Institute & Hull York Medical School, University of Hull, United Kingdom

## Abstract

**Background:**

Prospective cohort studies in relation to the associations between n-3 polyunsaturated fatty acids (PUFA) and risk of type 2 diabetes (T2D) were inconsistent. Differences in tissue n-3 PUFA compositions in subjects with and without T2D were also inconsistent in both cohort and case-control studies. We conducted a systematic review and meta-analysis of prospective cohort studies to examine the associations of fish and n-3 PUFA intake with T2D risk. The differences in tissue n-3 PUFA compositions in subjects with and without T2D were investigated based on cohort and case-control studies.

**Methods and Findings:**

PubMed, Embase, Cochrane library, China National Knowledge Infrastructure (CNKI) and Chinese VIP database up to January 2012 was used to identify relevant studies, and reference lists from retrieved studies were reviewed. Two authors independently extracted the data. Random-effects models were used to pool the summary relative risk (RR). Twenty-four studies including 24,509 T2D patients and 545,275 participants were identified. For cohort studies, the summary RR of T2D for the highest vs lowest categories of total fish, marine n-3 PUFA and alpha-linolenic acid intake was 1.07 (95% CI: 0.91, 1.25), 1.07 (95% CI: 0.95, 1.20) and 0.93 (95% CI: 0.81, 1.07), respectively. Subgroup analyses indicated that summary RR (highest vs lowest category) of T2D for fish and marine n-3 PUFA intake was 0.89 (95% CI: 0.81, 0.98) and 0.87 (95% CI: 0.79, 0.96) for Asian populations, and 1.20 (95% CI: 1.01, 1.44) and 1.16 (95% CI: 1.04, 1.28) for Western populations. Asian subjects with T2D had significantly lower tissue compositions of C22∶6n-3 (SMD: −1.43; 95% CI: −1.75, −1.12) and total n-3 PUFA (SMD: −1.41; 95% CI: −2.23, −0.59) compared with those without T2D.

**Conclusion:**

This systematic review and meta-analysis provides evidence that marine n-3 PUFA have beneficial effects on the prevention of T2D in Asian populations.

## Introduction

Type 2 diabetes (T2D) is one of the most common chronic diseases in the world, leading to a huge economic burden for society [1]. Dietary factors were postulated to play an important role in the prevention of T2D [2], [3]. N-3 polyunsaturated fatty acids (PUFA), especially marine n-3 PUFA (eicosapentaenoic acid (C20∶5n-3, EPA) and docosahexaenoic acid (C22∶6n-3, DHA)) intake, had been demonstrated to improve insulin sensitivity in animal models [4]. However, observational studies in relation to the association of n-3 PUFA intake with risk of T2D were inconsistent [3], [5]–[11]. In addition, fish, rich in marine n-3 PUFA, also showed inconsistent associations with risk of T2D in observational studies [2], [6]–[10], [12]–[15]. An ecological study of 41 countries revealed that fish and seafood intake might reduce the risk of T2D in populations with a high prevalence of obesity [12]. Fish consumption was also associated with lower risk of glucose intolerance from the Seven Countries Study [2] and an elderly population [13]. But prospective cohort studies had reported inverse [10], [14], [15], positive [6], [9], or null associations [7], [8] between fish intake and risk of T2D.

Tissue (plasma/serum/erythrocytes) n-3 PUFA compositions were reported to be significantly lower in subjects with T2D compared with control subjects in case-control studies [16], [17]. Nevertheless, many other case-control or cohort studies showed inconsistent results [5], [18]–[22].

Therefore, the aim of the present meta-analysis was to investigate the associations of fish and n-3 PUFA intake with risk of T2D based on prospective cohort studies. The differences of tissue (plasma/serum/erythrocytes) n-3 PUFA compositions in subjects with and without T2D were also investigated based on prospective cohort and case-control studies. Stratified analyses were conducted to examine sources of heterogeneity.

## Methods

### Search Strategy

Our report followed the Meta-analysis of Observational Studies in Epidemiology Guidelines [23]. Results were reported according to PRISMA guidelines (http://www.prisma-statement.org; Text S1). Our protocol was available in Text S2. PubMed, Embase, Cochrane library, China National Knowledge Infrastructure (CNKI) and Chinese VIP database up to January 2012 was searched. Following Medical Subject Headings terms or key words were searched: “fish” OR “fish oils” OR “seafood” OR “fatty acids” OR “docosahexaenoic acid” OR “eicosapentaenoic acid” OR “alpha-linolenic acid” AND “diabetes mellitus, type 2”. The search was restricted to human studies and had no language restriction. References from the retrieved articles were reviewed to identify potential bibliographies. Authors were not contacted for detailed information of primary studies.

### Selection Criteria

Two authors (JZ and TH) independently conducted the search and discrepancies were resolved through group discussion. To investigate the associations of fish and n-3 PUFA intake with risk of T2D, the inclusion criteria were: 1) prospective cohort study design; 2) the exposure of interest was dietary intake of fish, fatty fish, shellfish, n-3 PUFA, marine n-3 PUFA or alpha-linolenic acid (C18∶3n-3, ALA); 3) the endpoint of interest was T2D incidence; 4) relative risk (RR) or hazard ratio (HR) with the corresponding 95% confidence interval (CI) of T2D for each category of fish or n-3 PUFA intake were provided; and 5) if the same population or cohort was duplicated, the most recent and complete study was included. To investigate the differences in tissue (plasma/serum/erythrocytes) n-3 PUFA compositions in subjects with and without T2D, the inclusion criteria were: 1) prospective cohort or case-control study design; 2) tissue compositions of C22∶6n-3, C20∶5n-3, C18∶3n-3 or total n-3 PUFA in subjects with and without T2D were provided; 3) both the cases and controls in each study were from the same population. The exclusion criteria were: studies with cross-sectional, ecologic or intervention study design; duplicate studies; studies without detailed risk estimates of T2D for dietary fish or n-3 PUFA intake, or without tissue n-3 PUFA compositions in T2D subjects and controls.

### Data Extraction

The following information was extracted from the included studies: first author’s name, year of publication, study region and population, duration of follow-up, age of subjects, gender, number of events, participants and person-years for the entire study and for each fish or n-3 PUFA intake category, adjusted covariates, method of dietary assessment, RR or HR with their 95% CIs for each fish or n-3 PUFA intake category, tissue compositions of C22∶6n-3, C20∶5n-3, C18∶3n-3 and total n-3 PUFA in subjects with and without T2D. The greatest degree of adjusted RR or HR from each cohort study was extracted.

### Statistical Analysis

RR was taken as the common risk estimate for the associations between fish and n-3 PUFA intake and T2D risk. HR was considered as RR directly. RR from each study was firstly transformed to their natural logarithm and corresponding 95% CIs were used to calculate standard errors. DerSimonian and Laird random-effects model, which took both within- and between-study variation into consideration was used to combine the RRs and they were weighted by the inverse of their variances. The differences in tissue n-3 PUFA compositions in subjects with and without T2D were also analyzed as standardized mean difference (SMD) by pooling the data from case-control and cohort studies.

To further examine the relationship between fish intake and T2D risk, total fish consumption was standardized and categorized into four groups: 1) high (>5 servings/wk); 2) moderate (2–4 servings/wk); 3) low (1 serving/wk); and 4) reference group (<1 serving/wk or 1–3 servings/wk), if both <1 serving/wk and 1–3 servings/wk were available in a study, <1 serving/wk was chosen as reference group. Each RR from included studies was assigned into a corresponding standardized group. If more than one fish intake category fell into the same standardized group, the RRs were combined for further analysis. One portion or serving of fish was regarded as 105 g as described previously [24].

Dose-response analyses for total fish and marine n-3 PUFA intake were conducted according to Greenland and Longnecher [25] and Orsini et al [26], using a method described previously [24]. One study [14] which only reported two fish intake categories and two studies [5], [27] which did not report fish intake dose for each category were excluded from this analysis.

Statistical heterogeneity was assessed with the Q (significant at *P*<0.1) and *I^2^* statistics [28]. *I^2^* values of 25%, 50% and 75% correspond to cut-off points for low, moderate and high degrees of heterogeneity. Subgroup analyses were conducted to examine the sources of heterogeneity. Influence of individual study on the overall risk estimate or effect size was examined by sensitivity analysis in which one study at a time was excluded. Begg’s funnel plot and Egger’s regression test (significant at *P*<0.1) were used to evaluate publication bias. All the analyses were performed by using STATA version 11 (StataCorp LP, College Station, TX, USA).

## Results

### Literature Search

**Figure 1:**
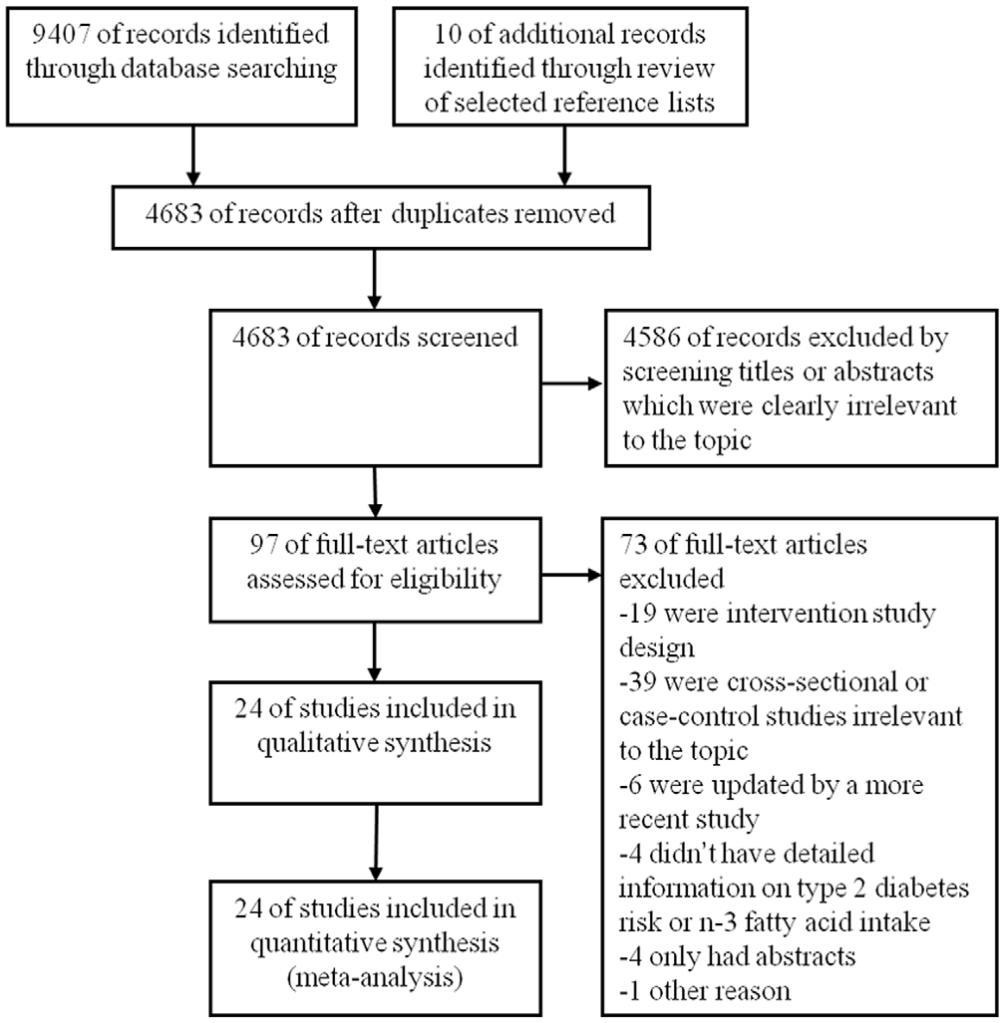
PRISMA flow diagram for selection of studies in the meta-analysis.

Twenty-four published studies [3], [5]–[11], [14]–[22], [27], [29]–[34] including 24,509 T2D patients and 545,275 participants were identified from a full-text examination of 97 potentially relevant studies (Figure 1), 1 study from Australia [5], 1 study from Cuba [19], 7 studies from Europe [7], [14], [17], [21], [22], [27], [33], 8 studies from Asia [10], [11], [15], [16], [30]–[32], [34], and 7 studies from the US [3], [6], [8], [9], [18], [20], [29]. Among the included studies, 10 studies [3], [5]–[11], [27], [29] reported the association between n-3 PUFA and T2D risk, 7 studies [6]–[10], [14], [15] reported the association between fish intake and T2D risk, and 5 studies [6]–[10] reported both the associations of n-3 PUFA and fish intake with T2D risk. Six studies [5], [8], [9], [11], [27], [29] assessed the association between C18∶3n-3 intake and risk of T2D. Thirteen studies [5], [16]–[22], [30]–[34] reported tissue n-3 PUFA compositions in subjects with and without T2D.

### Study Characteristics

A total of 23,226 T2D cases among 515,537 subjects were included in the meta-analysis of dietary n-3 PUFA and fish intake and T2D risk, with an average 9.9 years of follow-up (Table 1). For the analysis of different tissue n-3 PUFA compositions between subjects with and without T2D, 1629 T2D cases and 8727 controls were involved (Table 2). For one study [15], the RR for total fish intake was not available, RR for oily fish intake was taken as total fish intake directly. Two studies [10], [15] reported men and women separately, and each study was taken as 2 independent cohort studies of men and women. In another study [5], the relationship between C22∶6n-3 and C20∶5n-3 to T2D risk were reported separately, RRs from C22∶6n-3 and C20∶5n-3 were pooled for further analysis. Fatty acid compositions of plasma or serum PL were used for analysis if multiple tissue n-3 PUFA compositions were reported.

**Table 1:** Characteristics of included prospective cohort studies in the meta-analysis of dietary fish and n-3 polyunsaturated fatty acid intake and type 2 diabetes.

Study source	Duration of follow-up (years)	Age (y)	No. of cases/size of cohort	Fish or n-3 PUFA type	Exposure range (g/d)	Adjusted RR (95% CI)	Adjusted variables
Meyer et al, 2001 (3)	11	55–69	1890/35988	LC n-3 PUFA	Highest: 0.39; ref: 0.03	1.11 (0.94, 1.30)	Age, total energy, WHR, BMI, physical activity, cigarette smoking, alcohol consumption, education, marital status, residential area, hormone replacement therapy, energy-adjusted dietary magnesium and cereal fiber, dietary protein, saturated fatty acids, polyunsaturated fatty acids, monounsaturated fatty acids, *trans* fatty acids and cholesterol.
van Dam et al, 2002 (29)	12	40–75	1321/42504	C18∶3n-3	Highest: 0.671; ref: 0.321	0.93 (0.78, 1.11)	Age, total energy intake, time period, physical activity, cigarette smoking, alcohol consumption, hypercholesterolemia, hypertension, family history of type 2 diabetes, intake of cereal fiber and magnesium and BMI
Hodge et al, 2007 (5)	4	36–72	364/3737	C22∶6n-3	Highest: Q5; ref: Q1	0.77 (0.52, 1.16)	Age, sex, country of birth, family history of diabetes, physical activity, alcohol intake, BMI, and waist-hip ratio
				C20∶5n-3	Highest: Q5; ref: Q1	0.68 (0.62, 1.34)	
				C18∶3n-3	Highest: Q5; ref: Q1	1.14 (0.75, 1.73)	
Kaushik et al_Nurses’ Health Study, 2009 (6)	18	30–55	4159/61031	LC n-3 PUFA	Highest: 0.49; ref: 0.06	1.23 (1.11, 1.37)	Smoking, alcohol consumption, physical activity, family history of diabetes mellitus, BMI, intakes of saturated fat, trans fats, linolenic acid, linoleic acid, caffeine, cereal fiber, glycemic index, calories, menopausal status, and postmenopausal hormone use
				Fish	Highest: ≥5 times/wk; ref: <1 time/mo	1.29 (1.05, 1.57)	
Kaushik et al_Nurses’ Health Study 2, 2009 (6)	14	26–46	2728/91669	LC n-3 PUFA	Highest: 0.36; ref: 0.06	1.25 (1.10, 1.42)	Smoking, alcohol consumption, physical activity, family history of diabetes mellitus, BMI, intakes of saturated fat, trans fats, linolenic acid, linoleic acid, caffeine, cereal fiber, glycemic index, calories, use of hormone replacement therapy and oral contraceptive use
				Fish	Highest: ≥5 times/wk; ref: <1 time/mo	1.32 (0.99, 1.74)	
Kaushik et al_Health Professionals Follow-up Study, 2009 (6)	18	39–78	2493/42504	LC n-3 PUFA	Highest: 0.62; ref: 0.09	1.12 (0.98, 1.28)	Smoking, alcohol consumption, physical activity, family history of diabetes mellitus, BMI, intakes of saturated fat, trans fats, linolenic acid, linoleic acid, caffeine, cereal fiber, glycemic index, and calories
				Fish	Highest: ≥5 times/wk; ref: <1 time/mo	1.16 (0.96, 1.41)	
van Woudenbergh et al, 2009 (7)	15	≥55	463/4472	C22∶6n-3+ C20∶5n-3	Highest: 0.2368; ref: 0.0238	1.05 (0.80, 1.38)	Age, sex, smoking, education level, intake of energy, alcohol, trans fatty acid, fiber, intake of selenium, vitamin D and cholesterol
				Total fish	Highest: 35.6; ref: 0	1.32 (1.02, 1.70)	Age sex, smoking, education level, intake of energy, alcohol, trans fatty acid, and fiber
				Fatty fish	Highest: ≥15.7; ref: 0	0.99 (0.71, 1.38)	Age sex, smoking, education level, intake of energy, alcohol, trans fatty acid, fiber and lean fish
				Lean fish	Highest: 30.6; ref: 0	1.30 (1.01, 1.68)	Age sex, smoking, education level, intake of energy, alcohol, trans fatty acid, fiber and fatty fish
Patel et al, 2009 (14)	10.2	40–79	725/21984	Total fish	Highest: ≥1 portion/wk; ref: <1 portion/wk	0.75 (0.58, 0.96)	Age, sex, family history of diabetes, smoking, education level, physical activity, total energy intake, alcohol intake, plasma vitamin C, BMI, and waist circumference
				Oily fish	Highest: ≥1 portion/wk; ref: <1 portion/wk	0.94 (0.78, 1.13)	
				White fish	Highest: ≥1 portion/wk; ref: <1 portion/wk	0.87 (0.73, 1.03)	
				Shellfish	Highest: ≥1 portion/wk; ref: <1 portion/wk	1.36 (1.02, 1.81)	
Djousse et al_Women’s Health Study, 2011 (9)	12.4	≥45	2370/36328	Marine n-3 PUFA	Highest: 0.43; ref: 0.07	1.44 (1.25, 1.65)	Age, BMI, parental history of diabetes, smoking, exercise, alcohol intake, menopausal status, red-meat intake, and quintiles of energy intake, linoleic acid, α-linolenic acid, dietary magnesium, trans fat, saturated fat, cereal fiber, and glycemic index
				C18∶3n-3	Highest: 1.59; ref: 0.79	1.01 (0.85, 1.21)	
				Fish	Highest: 3.93 servings/wk; ref: 0.47 servings/wk	1.49 (1.30, 1.70)	
Djousse et al_Cardiovascular Health Study, 2011 (8)	10.6	≥65	204/3088	C22∶6n-3+ C20∶5n-3	Highest: >0.56; ref: ≤0.17	1.04 (0.67, 1.60)	Age, race, sex, clinic site, BMI, alcohol consumption, physical activity, current smoking, LDL cholesterol, and linoleic acid
				C18∶3n-3	Highest: >0.18; ref: ≤0.11	0.50 (0.24, 1.05)	
				Fish	Highest: ≥5/wk; ref: <1/mo	1.07 (0.35, 3.30)	Age, race, sex, clinic site, BMI, alcohol consumption, physical activity, current smoking, total energy intake and LDL cholesterol
Nanri et al_men, 2011 (15)	5	45–75	572/22921	Oily fish	Highest: 71.2; ref: 10.7	0.79 (0.59, 1.05)	Age, study area, BMI, smoking status, alcohol consumption, family history of diabetes mellitus, total physical activity, history of hypertension, total energy intake, coffee consumption, intake of calcium, magnesium, dietary fiber, vegetable, fruit, meat, and rice
				Lean fish	Highest: 30; ref: 3.3	1.05 (0.80, 1.38)	
Nanri et al_women, 2011 (15)	5	45–75	399/29759	Oily fish	Highest: 68.1; ref: 10.7	0.93 (0.67, 1.29)	The same as above
				Lean fish	Highest: 23.3; ref: 2.7	1.02 (0.75, 1.40)	
Villegas et al_men, 2011 (10)	4.1	40–74	900/51963	LC n-3 PUFA	Highest: 0.2; ref: 0.02	0.89 (0.70, 1.12)	Age, energy intake, waist-to-hip ratio, BMI, smoking, alcohol consumption, physical activity, income level, educational level, occupation, family history of diabetes, hypertension and dietary pattern
				Fish	Highest: 79; ref: 9.7	0.94 (0.74, 1.17)	
				shellfish	Highest: 24.3; ref: 1.6	0.82 (0.65, 1.02)	
Villegas et al_women, 2011 (10)	8.9	40–70	3034/64193	LC n-3 PUFA	Highest: 0.2; ref: 0.02	0.84 (0.74, 0.95)	The same as above
				Fish	Highest: 80.2; ref: 9.5	0.89 (0.78, 1.01)	
				shellfish	Highest: 23.5; ref: 1.4	0.86 (0.76, 0.99)	
Brostow et al, 2011 (11)	5.7	45–74	2252/43176	C22∶6n-3+ C20∶5n-3	Highest: 0.6; ref: 0.11	0.93 (0.77, 1.11)	Age, sex, dialect, year of interview, educational level, BMI, physical activity, smoking status, alcohol use, hypertension, intakes of omega-6, alternate omega-3, monounsaturated fat, saturated fat, dietary fiber, protein, and total energy
				C18∶3n-3	Highest: 1.06; ref: 0.27	0.79 (0.67, 0.93)	
Kroger et al, 2011 (27)	7	35–65	673/2724	LC n-3 PUFA	Highest: 0.59; ref: 0.04 (% of total fat intake)	1.29 (0.95, 1.75)	Age, sex, BMI, waist circumference, cycling, sports activity, education, smoking status, alcohol intake, occupational activity, coffee intake, fiber intake, total fat intake and energy intake
				C18∶3n-3	Highest: 2.6; ref: 1.4 (% of total fat intake)	1.13 (0.80, 1.59)	

Abbreviations: Q: quintile; ref: reference; LC n-3 PUFA: long-chain n-3 polyunsaturated fatty acids (C22∶6n–3+ C20∶5n-3).

**Table 2:** Characteristics of included studies in the meta-analysis of different n-3 polyunsaturated fatty acids compositions between subjects with and without type 2 diabetes.

Study source	Mean age(years)	Study design	No. of cases	No. of controls	Tissue n-3 PUFAs composition (% of totalfatty acids, case vs controls)
Faas et al, 1988 (20)	48.5	Case-control	5	5	Plasma C22∶6n-3 (1.6±0.7 vs 1.5±0.5)
					Red blood cell C22∶6n-3 (4.4±1.1 vs 4.4±1.1)
Bohov et al, 1993 (21)	60.6	Case-control	183	114	Serum C22∶6n-3 (1.96±0.05 vs 1.67±0.06)
					Serum C20∶5n-3 (0.66±0.03 vs 0.62±0.03)
					Serum C18∶3n-3 (0.42±0.01 vs 0.55±0.01)
					Serum n-3 PUFA (3.53±0.08 vs 3.31±0.09)
Pelikanova et al, 2001 (22)	40.4	Case-control	21	24	Serum PL C22∶6n-3 (3.04±0.87 vs 2.11±0.49)
					Serum PL C20∶5n-3 (1.04±0.54 vs 0.82±0.41)
					Serum PL C18∶3n-3 (0.20±0.19 vs 0.29±0.14)
					Serum PL n-3 PUFA (5.40±1.75 vs 4.67±1.39)
Rodriguez et al, 2004 (19)	53	Case-control	13	13	Plasma PL C22∶6n-3 (4.01±0.80 vs 3.47±0.89)
					Plasma PL C20∶5n-3 (0.64±0.23 vs 0.74±0.35)
					Plasma PL C18∶3n-3 (0.18±0.13 vs 0.20±0.13)
					Plasma PL n-3 PUFA (5.96±0.73 vs 5.49±1.17)
					Red blood cell PL C22∶6n-3 (3.9±1.35 vs 3.91±0.71)
					Red blood cell PL C20∶5n-3 (0.52±0.23 vs 0.44±0.12)
					Red blood cell PL C18∶3n-3 (0.15±0.04 vs 0.15±0.06)
					Red blood cell PL n-3 PUFA (7.18±1.71 vs 5.94±1.15)
Bakan et al, 2006 (34)	56.5	Case-control	32	20	Plasma C22∶6n-3 (1.9±0.9 vs 2.7±0.4)
Mao et al, 2007 (30)	57.4	Case-control	62	53	Serum PL C22∶6n-3 (5.1±1.3 vs 6.4±1.1)
					Serum PL C20∶5n-3 (1.9±0.6 vs 1.9±0.5)
					Serum PL C18∶3n-3 (0.32±0.2 vs 0.33±0.13)
					Serum PL n-3 PUFA (8.2±1.6 vs 9.6±1.5)
Krachler et al, 2008 (17)	51.6	Case-control	159	291	Erythrocyte membrane C22∶6n-3 (4.61±1.01 vs 4.83±1.02)
					Erythrocyte membrane C20∶5n-3 (1.31±0.45 vs 1.37±0.46)
					Erythrocyte membrane C18∶3n-3 (0.35±0.10 vs 0.36±0.13)
Lou et al, 2010 (31)	56.1	Case-control	60	55	Serum PL C22∶6n-3 (4.11±1.32 vs 6.41±1.26)
					Serum PL C20∶5n-3 (1.80±0.55 vs 1.90±0.52)
					Serum PL C18∶3n-3 (0.28±0.08 vs 0.33±0.10)
					Serum PL n-3 PUFA (6.08±1.66 vs 9.54±1.54)
Huang et al, 2010 (16)	60	Case-control	180	186	Plasma PL C22∶6n-3 (2.46±2.2 vs 5.8±2.0)
					Plasma PL C20∶5n-3 (0.99±0.5 vs 2.12±0.7)
					Plasma PL C18∶3n-3 (0.36±0.1 vs 0.70±0.2)
					Plasma PL n-3 PUFA (4.52±2.8 vs 9.22±1.8)
Zhang et al, 2011 (32)	49.2	Case-control	241	156	Plasma total n-3 PUFA (5.52±0.77 vs 6.47±2.27)
Vessby et al, 1994 (33)	50	Prospective cohort	75	1753	Serum CE C22∶6n-3 (0.68±0.21 vs 0.70±0.21)
					Serum CE C20∶5n-3 (1.42±0.57 vs 1.35±0.63)
					Serum CE C18∶3n-3 (0.65±0.18 vs 0.66±0.16)
Wang et al, 2003 (18)	52	Prospective cohort	252	2657	Plasma PL C22∶6n-3 (2.71±0.83 vs 2.76±0.84)
					Plasma PL C20∶5n-3 (0.58±0.33 vs 0.56±0.31)
					Plasma PL C18∶3n-3 (0.13±0.05 vs 0.15±0.05)
					Plasma CE C22∶6n-3 (0.43±0.15 vs 0.43±0.15)
					Plasma CE C20∶5n-3 (0.59±0.36 vs 0.54±0.28)
					Plasma CE C18∶3n-3 (0.4±0.11 vs 0.42±0.11)
Hodge et al, 2007 (5)	56.2	Prospective cohort	346	3391	Plasma PL C22∶6n-3 (4.15±0.99 vs 4.02±1.07)
					Plasma PL C20∶5n-3 (1.15±0.50 vs 1.05±0.47)
					Plasma PL C18∶3n-3 (0.17±0.08 vs 0.17±0.08)
					Plasma PL n-3 PUFA (6.77±1.36 vs 6.55±1.40)

Abbreviations: n-3 PUFAs: n-3 polyunsaturated fatty acids; PL: phospholipids; CE: cholesterol esters.

### Fish Intake and Risk of T2D

**Figure 2:**
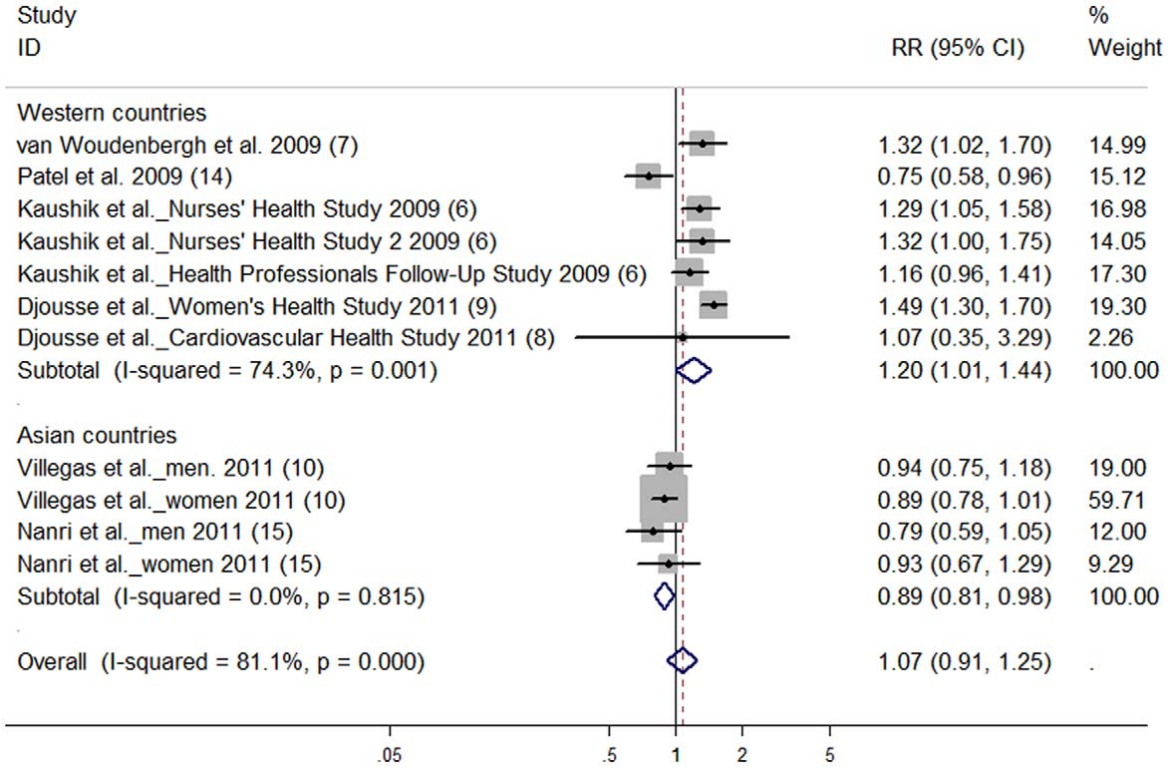
Relative risk of type 2 diabetes for highest vs lowest categories of total fish intake. The combined relative risk was achieved using random-effects model. Grey square represents relative risk in each study, with square size reflecting the study-specific weight and the 95% CI represented by horizontal bars. The diamond indicates summary risk estimate.

There was no significant association between total fish intake (highest vs lowest category) and risk of T2D (RR: 1.07; 95% CI: 0.91, 1.25) (Figure 2). High degree of heterogeneity was observed (*P* for heterogeneity <0.001, *I^2^* = 81.1). No publication bias was observed from Begg’s funnel plot and Egger’s test (*P* = 0.60).

The summary RR of T2D was 1.03 (95% CI: 0.90, 1.19), 1.06 (95% CI: 0.90, 1.25), and 1.05 (95% CI: 0.97, 1.13) for high (>5 per wk), moderate (2–4 per wk), and low (1 per wk) fish intake respectively (Table 3). No dose-response association for total fish intake and risk of T2D was observed (RR: 1.02; 95% CI: 0.98, 1.06).

**Table 3:** Subgroup analyses for the associations of fish intake with risk of type 2 diabetes according to standardized fish intake categories (high, moderate and low).

Fish intake classification	No. of cohorts	RR (95% CI)	*P*-heterogeneity	*I* ^2^ (%)
**High fish intake (>5 per wk)**	8	1.03 (0.9, 1.19)	0.011	61.6
Regions				
Asian countries	4	0.89 (0.81, 0.98)	0.815	0
Western countries	4	1.24 (1.09, 1.40)	0.834	0
US	4	1.24 (1.09, 1.40)	0.834	0
Follow-up duration				
>9.9 years	4	1.24 (1.09, 1.40)	0.834	0
≤9.9 years	4	0.89 (0.81, 0.98)	0.815	0
Gender				
Men	3	0.97 (0.78, 1.21)	0.077	61.1
Women	4	1.08 (0.86, 1.36)	0.005	77
Both	1	1.07 (0.35, 3.29)		
**Moderate fish intake (2–4 per wk)**	10	1.06 (0.90, 1.25)	<0.001	89
Regions				
Asian countries	4	0.83 (0.78, 0.89)	0.934	0
Western countries	6	1.25 (1.12, 1.40)	0.067	51.4
Europe	1	1.32 (1.02, 1.70)		
US	5	1.24 (1.09, 1.42)	0.038	60.7
Follow-up duration				
>9.9 years	6	1.25 (1.12, 1.40)	0.067	51.4
≤9.9 years	4	0.83 (0.78, 0.89)	0.934	0
Gender				
Men	3	0.94 (0.78, 1.13)	0.038	69.4
Women	5	1.10 (0.84, 1.44)	<0.001	94.2
Both	2	1.28 (1.00, 1.64)	0.434	0
**Low fish intake (1 per wk)**	10	1.05 (0.97, 1.13)	0.016	55.8
Regions				
Asian countries	4	0.96 (0.88, 1.04)	0.92	0
Western countries	6	1.11 (1.03, 1.20)	0.121	42.6
Europe	1	1.19 (0.92, 1.54)		
US	5	1.10 (1.01, 1.20)	0.073	53.2
Follow-up duration				
>9.9 years	6	1.11 (1.03, 1.20)	0.121	42.6
≤9.9 years	4	0.96 (0.88, 1.04)	0.92	0
Gender				
Men	3	0.97 (0.88, 1.06)	0.812	0
Women	5	1.08 (0.98, 1.20)	0.01	69.8
Both	2	1.17 (0.92, 1.48)	0.646	0

The summary risk estimates for the highest vs lowest categories of fatty fish, lean fish and shellfish intake were 0.91 (95% CI: 0.80, 1.04), 1.03 (95% CI: 0.86, 1.24) and 0.96 (95% CI: 0.75, 1.25) respectively. No publication bias was observed for all the analyses of fatty fish, lean fish and shellfish intake (data not shown).

### N-3 PUFA Intake and Risk of T2D

**Figure 3:**
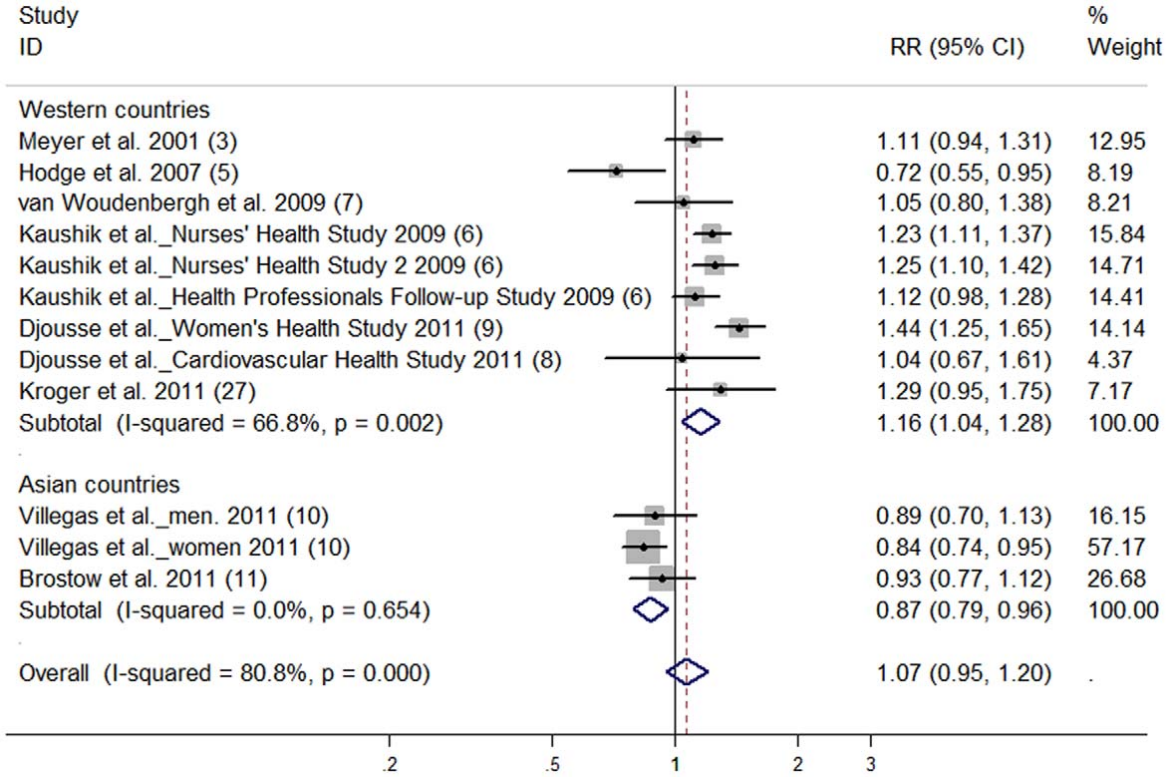
Relative risk of type 2 diabetes for highest vs lowest categories of marine n-3 polyunsaturated fatty acids intake. The combined relative risk was achieved using random-effects model. Grey square represents relative risk in each study, with square size reflecting the study-specific weight and the 95% CI represented by horizontal bars. The diamond indicates summary risk estimate.

There was no significant association between marine n-3 PUFA intake and risk of T2D (highest vs lowest category; RR: 1.07; 95% CI: 0.95, 1.20) (Figure 3). High degree of heterogeneity was found (*P* for heterogeneity <0.001, *I^2^* = 80.8). Overall, no publication bias was observed from Begg’s funnel plot and Egger’s test (*P* = 0.338). No significant dose-response association between marine n-3 PUFA and risk of T2D was found (RR: 1.01; 95% CI: 0.98, 1.05).

**Figure 4:**
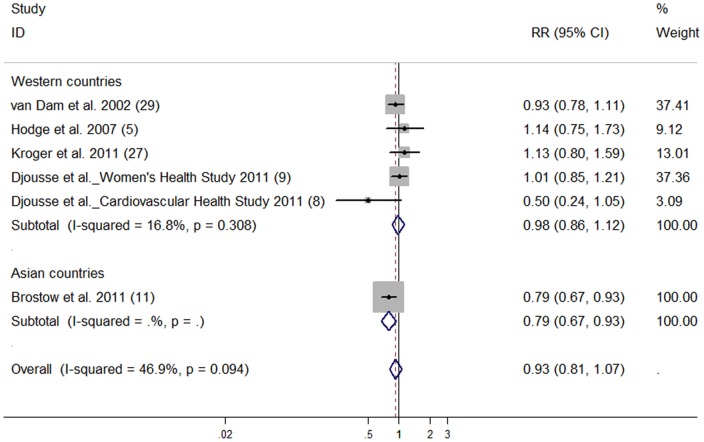
Relative risk of type 2 diabetes for highest vs lowest categories of alpha-linolenic acid intake. The combined relative risk was achieved using random-effects model. Grey square represents relative risk in each study, with square size reflecting the study-specific weight and the 95% CI represented by horizontal bars. The diamond indicates summary risk estimate.

The pooled RR of T2D was 0.93 (95% CI: 0.81, 1.07) for the highest vs lowest C18∶3n-3 intake (Figure 4). No publication bias was observed (data not shown).

### Differences in Tissue n-3 PUFA Compositions between Subjects with and without T2D

Subjects with T2D had significantly lower tissue compositions of C18∶3n-3 (SMD: −0.48; 95% CI: -0.86, −0.11) compared with subjects without T2D. No publication bias was observed (data not shown). No significant differences were observed for the other n-3 PUFA compositions (Table 4).

**Table 4:** Subgroup analyses on different tissue n-3 polyunsaturated fatty acid compositions in subjects with and without type 2 diabetes.

N-3 PUFAs	No. of studies	SMD (95% CI)	*P*-heterogeneity	*I* ^2^ (%)
C22∶6n-3	12	−0.31 (−0.70, 0.07)	<0.001	96.4
Asian countries	4	−1.43 (−1.75, −1.12)	0.051	61.3
Western countries	8	0.16 (−0.05, 0.37)	<0.001	82.8
C20∶5n-3	10	−0.16 (−0.53, 0.21)	<0.001	96.3
Asian countries	4	−0.69 (−1.96, 0.59)	<0.001	97.9
Western countries	6	0.09 (−0.03, 0.20)	0.055	51.3
C18∶3n-3	10	−0.48 (−0.86, −0.11)	<0.001	96.5
Asian countries	3	−0.92 (−2.27, 0.42)	<0.001	98.0
Western countries	7	−0.27 (−0.50, −0.05)	<0.001	87.2
Total n-3 PUFA	8	−0.56 (−1.22, 0.09)	<0.001	98.0
Asian countries	4	−1.41 (−2.23, −0.59)	<0.001	96.7
Western countries	4	0.18 (0.08, 0.28)	0.638	0

Abbreviations: n-3 PUFAs: n-3 polyunsaturated fatty acids; SMD: standardized mean difference.

### Sensitivity Analyses

Subgroup analyses (Table 5) revealed that summary RR of T2D for the highest vs lowest total fish intake categories was 0.89 (95% CI: 0.81, 0.98) for studies in Asian populations, and 1.20 (95% CI: 1.01, 1.44) for studies in Western populations. Exclusion of two case-cohort studies produced similar results (RR: 1.10; 95% CI: 0.98, 1.23) as the overall risk estimate. Exclusion of one study [15] in which oily fish intake was considered as total fish intake directly did not greatly change the pooled RR (1.12; 95% CI: 0.94, 1.33). Subgroup analyses (Table 3) for high, moderate and low fish consumption models produced similar results as those derived from the relationship between fish intake and T2D risk (highest vs lowest category). Subgroup analyses (Table 5) found that for marine n-3 PUFA intake, summary RR of T2D for studies conducted in the Western populations (1.16; 95% CI: 1.04, 1.28) was higher than that in Asian populations (0.87; 95% CI: 0.79, 0.96).

**Table 5:** Subgroup analyses on fish and marine n-3 polyunsaturated fatty acids intake and risk of type 2 diabetes.

	Fish intake and risk of T2D				Marine n-3 PUFA (C22∶6n-3+ C20∶5n-3) and risk of T2D			
Group	No. of cohorts	RR (95% CI)	*P*-heterogeneity	*I* ^2^	No. of cohorts	RR (95% CI)	*P*-heterogeneity	*I* ^2^ (%)
All studies	11	1.07 (0.91, 1.25)	<0.001	81.1	12	1.07 (0.95, 1.20)	<0.001	80.8
Regions								
Asian countries	4	0.89 (0.81, 0.98)	0.815	0	3	0.87 (0.79, 0.96)	0.654	0
Western countries	7	1.20 (1.01, 1.44)	0.001	74.3	9	1.16 (1.04, 1.28)	0.002	66.8
Europe	2	1.00 (0.57, 1.73)	0.002	89.5	2	1.15 (0.94, 1.41)	0.324	0
US	5	1.33 (1.20, 1.48)	0.302	17.6	6	1.22 (1.13, 1.33)	0.109	44.5
Follow-up duration								
>9.9 years	7	1.20 (1.01, 1.44)	0.001	74.3	7	1.21 (1.12, 1.31)	0.116	41.2
≤9.9 years	4	0.89 (0.81, 0.98)	0.815	0	5	0.90 (0.78, 1.04)	0.061	55.5
Gender								
Men	3	0.97 (0.78, 1.21)	0.077	61.1	2	1.02 (0.82, 1.27)	0.096	64
Women	5	1.17 (0.91, 1.49)	<0.001	87.9	5	1.16 (0.97, 1.38)	<0.001	89.4
Both	3	1.00 (0.63, 1.61)	0.008	79.1	5	0.97 (0.81, 1.17)	0.075	53

Abbreviations: n-3 PUFA: n-3 polyunsaturated fatty acids.

**Figure 5:**
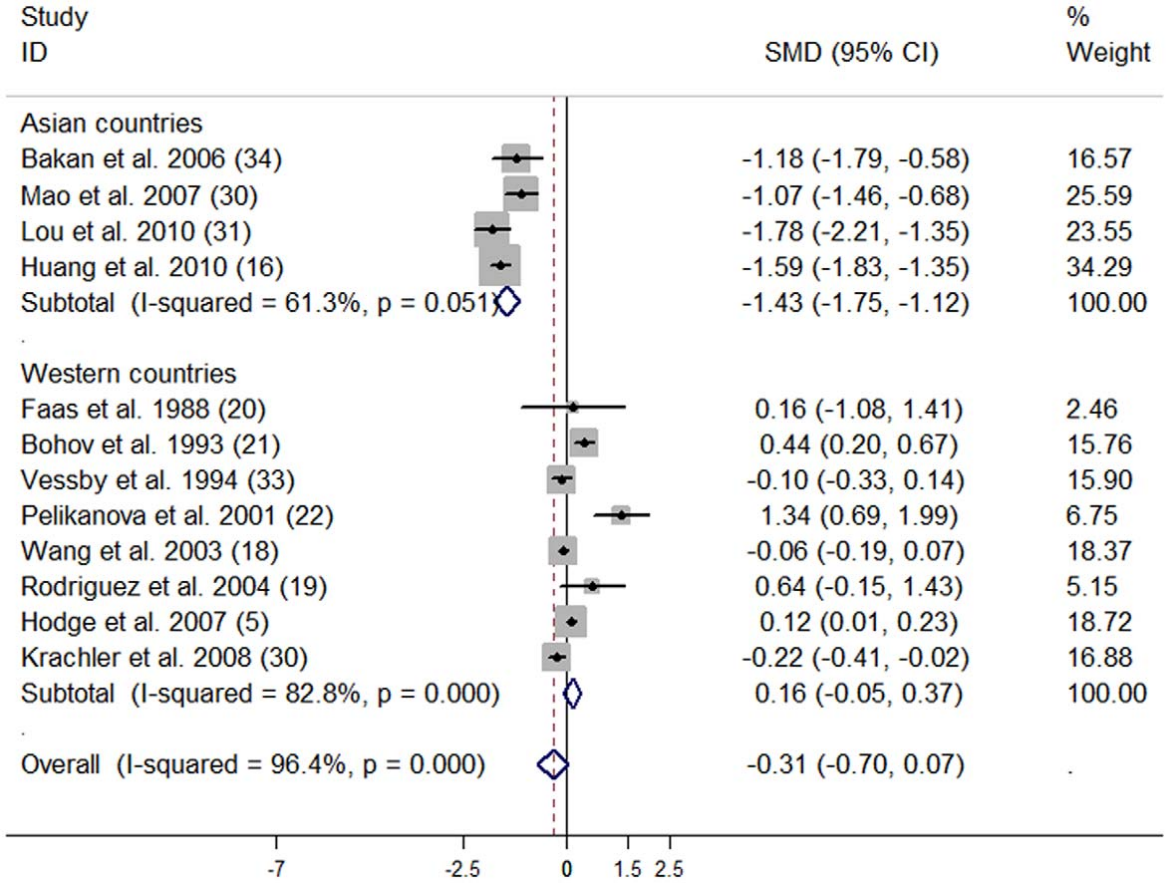
Effect of type 2 diabetes on tissue C22∶6n-3 composition compared with controls. The combined standardized mean difference (SMD) was achieved using random-effects model. Grey square represents SMD in each study, with square size reflecting the study-specific weight and the 95% CI represented by horizontal bars. The diamond indicates summary SMD.

**Figure 6:**
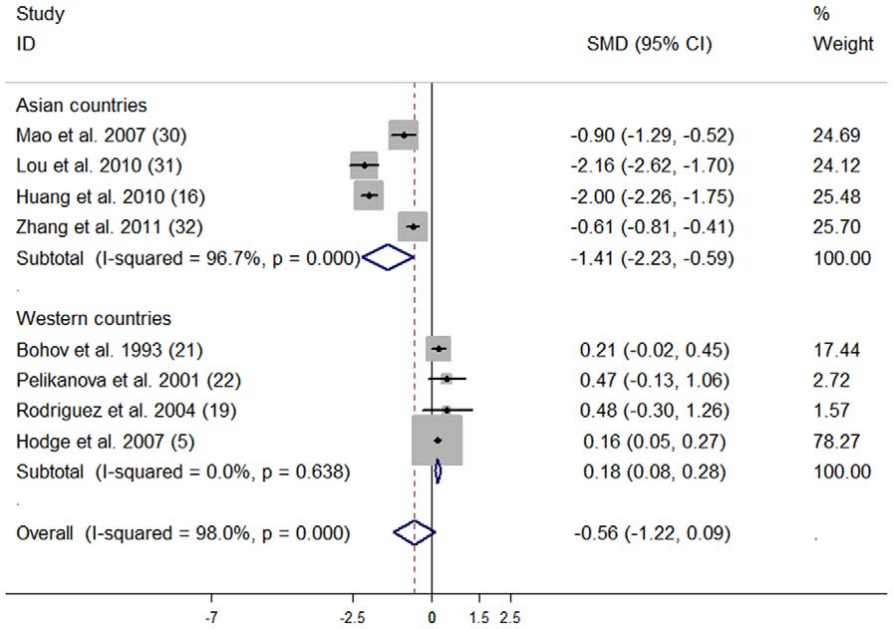
Effect of type 2 diabetes on tissue total n-3 PUFA composition compared with controls. The combined standardized mean difference (SMD) was achieved using random-effects model. Grey square represents SMD in each study, with square size reflecting the study-specific weight and the 95% CI represented by horizontal bars. The diamond indicates summary SMD.

Subgroup analyses (Table 4 **)** showed that Asian subjects with T2D compared with those without T2D, tissue compositions of C22∶6n-3 (SMD: −1.43; 95% CI: −1.75, −1.12) and total n-3 PUFA (SMD: −1.41; 95% CI: −2.23, −0.59) were significantly lower. (Figure 5, Figure 6). In Western populations, tissue compositions of total n-3 PUFA (SMD: 0.18; 95% CI: 0.08, 0.28) were significantly higher in subjects with T2D compared with controls; but tissue C18∶3n-3 was significantly lower (SMD: −0.27; 95% CI: −0.50, −0.05) in subjects with T2D compared with controls.

## Discussion

Overall, there were no significant associations between fish and n-3 PUFA intake and risk of T2D. However, subgroup analyses revealed that study regions remarkably affected the summary risk estimates and reduced the study heterogeneity. Marine n-3 PUFA and fish intake was inversely associated with risk of T2D only in Asian populations. Tissue C22∶6n-3 and total n-3 PUFA compositions were significantly lower in T2D subjects compared with controls in Asian but not Western populations.

One possible explanation was the influence of genetics on incidence of T2D as suggested by the high levels of differentiation between different populations for the ensemble of T2D loci [35] and the influences of gene-diet interaction [36], [37]. Klimentidis *et al*. [35] suggested that East Asians and sub-Saharan Africans experienced natural selection at loci associated with T2D, and there might be an evolutionary genetic basis for population differences in T2D. Compared with other common diseases, T2D risk alleles showed extreme directional differentiation across different populations, and the frequencies of T2D risk alleles showed a consistent decrease from Sub-Saharan African, through European, to East Asian populations. The predicted genetic risks of T2D also showed a significant difference across different populations, with lower risk in the Asian and higher risk in the African populations [38]. The disparities in T2D rates may reflect the adaptation of humans to the local environments along with human migration [39]. This is further supported by genome-wide association studies (GWAS)-identified genetic loci associated with T2D were substantially different between East Asian and European populations [40]. For example, genetic variants at some genes (*ADAMTS9*, *BCL11A*, *DCD*, *CENTD2*, *CHCHD2P9*, *DUSP9*, *FTO*, *JAZF1*, *PPARG*, *etc.*) associated with T2D only in the GWAS of European populations, while genetic variants at some others genes (*ANK1*, *CMIP*, *CR2*, *GCC1*, *GLIS3*, *HUNK*, *etc*.) associated with T2D only in the GWAS of East Asian populations [40]. This suggests that genetic backgrounds for the risk of T2D are different between different populations. Given different T2D loci or frequencies of risk alleles between East Asian and European populations, these two populations may respond differently to the same marine n-3 PUFA exposure in relation to the incidence of T2D. It is biologically plausible. Marine n-3 PUFA were known to affect gene expression through regulation of two group of transcription factors, including sterol regulatory element binding proteins and peroxisome proliferator-activated receptors (PPAR), both are crucial for the modulation of numerous gene expressions, including genes involved in the inflammation, lipid metabolism, energy utilization and insulin signaling [41], [42]. For example, genetic variants at *PPARG* associated with T2D only in the GWAS of Europeans, but not East Asians [40]. Expression of the *PPARG* can be regulated by n-3 PUFA [41], [42], and it is biologically plausible for the interaction of n-3 PUFA with *PPARG* variants to influence T2D risk in the European populations, but not in the East Asian populations. Therefore, as most of our included Asian studies are from East Asia, it is reasonable to postulate that n-3 PUFA intake may interact with a number of T2D-related genes for the risk of T2D, and there may be a different interaction pattern for n-3 PUFA and the T2D-related genes for the risk of T2D in the East Asian and Caucasian populations [35], [38]. However, genetic information in relation to the T2D-related variants was not available in most of these prospective studies, making it difficult to examine this hypothesis. In Asia, Huang *et al.* found that plasma n-3 PUFA were inversely associated with insulin sensitivity and metabolic syndrome in Chinese populations [16], [43]. Prospective studies also supported that for Chinese populations in Shanghai and in Singapore [10], [11], n-3 PUFA was inversely associated with incidence of T2D. In contrast, many studies from the US and Europe showed null [29] or even positive [3], [6], [9] association between n-3 PUFA and incidence of T2D. All these differences between Asian and US or European studies shed light on the possibility that gene-diet interaction might be an important explanation for the observed inconsistent association between n-3 PUFA and T2D incidence.

In addition to the influence of gene-diet interaction, differences in dietary patterns between Asian and Western populations might be another explanation for the inconsistent findings. Western dietary pattern is characterized by high intakes of sugar, red meat and fried food; while Asian dietary pattern, especially Chinese and Japanese, also known as a prudent dietary pattern, includes high intake of fruit, vegetable, fish and tofu. Western dietary pattern score was positively associated with risk of T2D, while a prudent dietary pattern score was inversely associated with risk of T2D [44], [45]. Fish intake is only part of the respective dietary pattern in different regions. Fried fish may be more popular in the Western populations, while Asians prefers boiled or steamed fish. Therefore, the different dietary patterns and their corresponding cooking methods may have been related to the inconsistent association of fish intake with T2D risk in the Western and Asian populations.

The potential benefit of marine n-3 PUFA is their incorporation into cell membranes, subsequently increasing insulin sensitivity [46]. Animal studies also supported the biological effect of marine n-3 PUFA on insulin resistance [4], [47]. However, in a meta-analysis of 26 randomized controlled trials [48], fish oil supplementation only marginally increased fasting blood glucose by 0.43 mmol/L in T2D patients. Another recent meta-analysis [49] found that n-3 PUFA intervention had no effects on insulin sensitivity compared to placebo. Most controlled trials [50], [51] did not find significant associations between fish oil treatment and parameters of glucose metabolism; some controlled trials [52], [53] even reported mild adverse effects of fish oil on glucose metabolism. However most of these studies were conducted in Western countries; and in the light of the previous hypothesis, genetic backgrounds and gene-diet interaction of populations in these countries may contribute to the contradictory results, and more randomized controlled trials with regard to fish oil supplement and glucose homeostasis in Asian populations are needed to confirm the hypothesis. In addition, the different response of tissue n-3 PUFA compositions to T2D in Asian and Western populations also indicated the existence of possible gene-diet interaction and genetic differences in the two regions.

The effects of fish consumption on T2D were highly consistent with that of marine n-3 PUFA in the present meta-analysis. Fish are rich sources of marine n-3 PUFA and the effects of fish on T2D might be attributed to the effects of C22∶6n-3 and C20∶5n-3. However, oily fish and lean fish, which contained quite different concentrations of C22∶6n-3 and C20∶5n-3, did not exert significantly different effects on risk of T2D compared with each other, and with total fish consumption in the present study; it might be that the study number for fatty fish and lean fish intake with T2D risk was small [7], [14], [15] and more research in this field is needed in future.

In contrast to marine n-3 PUFA, only a few cohort studies reported the association between C18∶3n-3 intake and T2D risk; further, C18∶3n-3 was lower in T2D subjects compared with controls, especially in Western populations. It might be that C18∶3n-3, the vegetable fatty acid, might affect the risk of T2D through different mechanisms compared with marine n-3 PUFA, which merits further investigations [54].

There are several strengths in the present study. First, large sample size of the included studies makes this meta-analysis more powerful to examine the associations between fish and n-3 PUFA intake and risk of T2D than any individual study. Second, although randomized controlled trials are the best way to achieve causal inference, however, there is no data for the association of fish and n-3 PUFA intake with T2D risk available in the literatures. Therefore, we systematic reviewed results from available cohort studies, and prospective nature of the included cohort studies (fish and marine n-3 PUFAs intake and T2D risk) makes the results less likely to be affected by recall and selection biases. Third, both the associations of marine and non-marine n-3 PUFA with risk of T2D were examined; and the associations of total fish, fatty fish, lean fish and shellfish intake with risk of T2D were all analyzed in this study, thus giving a relatively complete view of the current available evidence in relation to the associations of fish, n-3 PUFA intake with risk of T2D. Lastly, tissue n-3 PUFA compositions in subjects with and without T2D were compared, which supported the results from cohort studies.

Some limitations presented in this meta-analysis. First, classifications of fish and n-3 PUFA intake amounts were inconsistent among studies, which might have influenced the results. However, we used RRs of the highest vs lowest categories of fish or n-3 PUFA intake, which might reduce the bias caused by misclassification. Second, observational studies could not avoid residual confounders. Potential confounders always existed although most of the included studies had adjusted a wide range of confounders.

In conclusion, this systematic review and meta-analysis provided evidence that marine n-3 PUFA consumption had protective associations with risk of T2D in Asian populations, but was positively associated with risk of T2D in Western populations. T2D subjects in Asian but not in Western countries had lower tissue C22∶6n-3 and total n-3 PUFA compositions than controls. These findings have important public health implications. The prevalence of T2D has been steadily increasing around the world; dietary marine n-3 PUFA intake has proven to be protective for cardiovascular disease, but its effects on T2D risk were inconsistent among different populations. Influences of genetics and gene-diet interaction on T2D within different populations should be further explored to understand the association between marine n-3 PUFA intake and risk of T2D.

## Supporting Information

Text S1 **PRISMA checklist.**
(DOC)

Text S2 **Study protocol for systematic review and meta-analysis to determine the effects of n-3 polyunsaturated fatty acids on risk of type 2 diabetes.**
(DOC)
