# Supplementary material for: Marine N-3 Polyunsaturated Fatty Acids Are Inversely Associated with Risk of Type 2 Diabetes in Asians: A Systematic Review and Meta-Analysis
Source: PLoS One. 2012 Sep 11;7(9):e44525. doi: 10.1371/journal.pone.0044525 (PMC3439396; doi:10.1371/journal.pone.0044525)
Supplement: Text S2 — Study protocol for systematic review and meta-analysis to determine the effects of n-3 polyunsaturated fatty acids on risk of type 2 diabetes. (DOC) [file pone.0044525.s002.doc]

**Text S2 Study protocol for systematic review and meta-analysis to determine the effects of n-3 polyunsaturated fatty acids on risk of type 2 diabetes**

**Objective**

To investigate the associations of fish and n-3 PUFA intake with risk of T2D based on prospective cohort studies. The differences of tissue (plasma/serum/erythrocytes) n-3 PUFA compositions in subjects with and without T2D will also be investigated based on prospective cohort and case-control studies.

**Inclusion Criteria**

To investigate the associations of fish and n-3 PUFA intake with risk of T2D, the inclusion criteria are:

1) prospective cohort study design;

2) the exposure of interest is dietary intake of fish, fatty fish, shellfish, n-3 PUFA, marine n-3 PUFA or alpha-linolenic acid (C18:3n-3, ALA);

3) the endpoint of interest is T2D incidence;

4) relative risk (RR) or hazard ratio (HR) with the corresponding 95% confidence interval (CI) of T2D for each category of fish or n-3 PUFA intake are provided;

5) if the same population or cohort is duplicated, the most recent and complete study will be included.

To investigate the differences in tissue (plasma/serum/erythrocytes) n-3 PUFA compositions in subjects with and without T2D, the inclusion criteria are:

1) prospective cohort or case-control study design;

2) tissue compositions of C22:6n-3, C20:5n-3, C18:3n-3 or total n-3 PUFA in subjects with and without T2D are provided;

3) both the cases and controls in one study are from the same population.

**Search Methods**

We will search the following databases:

1) PubMed

2) Embase

3) Cochrane library

4) China National Knowledge Infrastructure (CNKI)

5) Chinese VIP database

There will be no restriction on language or year of publication

Following Medical Subject Headings terms or key words are searched:

“fishes” OR

“fish oils” OR

“seafood” OR

“fatty acids”OR

“docosahexaenoic acid” OR

“[eicosapentaenoic acid](http://www.ncbi.nlm.nih.gov/mesh/68015118)” OR

“alpha-linolenic acid” AND

“diabetes mellitus, type 2”.

The search will be restricted to human studies and have no language restriction. References from the retrieved articles will be reviewed to identify potential bibliographies.

**Data Collection**

Two authors (JZ and TH) will independently conduct the search and discrepancies will be resolved through group discussion.

The following information will be extracted from the included studies: first author’s name, year of publication, study region and population, duration of follow-up, age of subjects, gender, number of events, participants and person-years for the entire study and for each fish or n-3 PUFA intake category, adjusted covariates, method of dietary assessment, RR or HR with their 95% CIs for each fish or n-3 PUFA intake category, tissue compositions of C22:6n-3, C20:5n-3, C18:3n-3 and total n-3 PUFA in subjects with and without T2D. The greatest degree of adjusted RR or HR from each cohort study will be extracted.

**Meta-analysis**

RR will be taken as the common risk estimate for the associations between fish and n-3 PUFA intake and T2D risk. HR will be considered as RR directly. RR from each study will be firstly transformed to their natural logarithm and corresponding 95% CIs are used to calculate standard errors. DerSimonian and Laird random-effects model, which took both within- and between-study variation into consideration will be used to combine the RRs and they are weighted by the inverse of their variances. The differences in tissue n-3 PUFA compositions in subjects with and without T2D will also be analyzed as standardized mean difference (SMD) by pooling the data from case-control and cohort studies.

To further examine the relationship between fish intake and T2D risk, total fish consumption will be standardized and categorized into four groups: 1) high (>5 servings /wk); 2) moderate (2-4 servings /wk); 3) low (1 serving /wk); and 4) reference group (<1 serving /wk or 1-3 servings /wk), if both <1 serving /wk and 1-3 servings /wk are available in a study, <1 serving /wk will be chosen as reference group. Each RR from included studies will be assigned into a corresponding standardized group. If more than one fish intake category fall into the same standardized group, the RRs will be combined for further analysis. One portion or serving of fish will be regarded as 105 g as described previously .

Dose-response analyses for total fish and marine n-3 PUFA intake will be conducted according to Greenland and Longnecher and Orsini et al.

Statistical heterogeneity will be assessed with the Q (significant at *P* < 0.1) and *I^2^* statistics ; Subgroup analyses will be conducted to examine the sources of heterogeneity. Influence of individual study on the overall risk estimate or effect size will be examined by sensitivity analysis in which one study at a time is excluded. Begg’s funnel plot and Egger’s regression test (significant at *P* < 0.1) will be used to evaluate publication bias.

**References**

1. Zheng J, Huang T, Yu Y, Hu X, Yang B, et al. (2011) Fish consumption and CHD mortality: an updated meta-analysis of seventeen cohort studies. Public Health Nutr 15: 725-737.

2. Greenland S, Longnecker MP (1992) Methods for trend estimation from summarized dose-response data, with application to meta-analysis. Am J Epidemiol 135: 1301-1309.

3. Orsini N, Bellocco R, Greenland S (2006) Generalized least squares for trend estimation of summarized dose-response data. Stata J 6: 40-57.

4. Higgins JP, Thompson SG, Deeks JJ, Altman DG (2003) Measuring inconsistency in meta-analyses. BMJ 327: 557-560.
